# Normative need for orthodontic treatment and perception of the need for such treatment among Brazilian adolescents

**DOI:** 10.1590/2177-6709.22.3.041-046.oar

**Published:** 2017

**Authors:** Ana Karla de Almeida Pinto Monteiro, Dmitry José de Santana Sarmento, Tássia Cristina de Almeida Pinto-Sarmento, Michele Baffi Diniz, Ana Flávia Granville-Garcia, Danilo Antônio Duarte

**Affiliations:** 1 Universidade Estadual do Rio Grande do Norte, Department of Dentistry (Caico/RN, Brazil).; 2 Universidade de São Paulo, School of Dentistry, Division of Pathology, Department of Stomatology (São Paulo/SP, Brazil).; 3 Universidade Federal de Campina Grande, Academic Unit of Biological Sciences (Patos/PB, Brazil).; 4 Universidade Cruzeiro do Sul, Department of Dentistry (São Paulo/SP, Brazil).; 5 Universidade Estadual da Paraíba, Department of Dentistry (Campina Grande/PB, Brasil).

**Keywords:** Aesthetics, dental, Perception, Dental health surveys.

## Abstract

**Objective:**

The aim of the present study was to evaluate the normative need for orthodontic treatment and the perception of such need among students aged 11-14 years.

**Methods:**

A cross-sectional study was conducted with a sample of 346 students, randomly selected from public and private schools. The Dental Aesthetic Index (DAI) was used to evaluate the normative need for orthodontic treatment (NNOT). The perceived need for treatment among students was assessed by a previously validated questionnaire. Data was analyzed by Pearson’s Chi-squared test (α = 5%).

**Results:**

The prevalence of malocclusion and NNOT was 65.6%. Of the sample, 73.7% felt they needed treatment, 66.2% wanted orthodontic treatment and 62.7% were satisfied with their dental aesthetics. A statistically significant association was observed between NNOT and the perception of the need for treatment among the students (*p* < 0.001).

**Conclusion:**

The present study demonstrated a high prevalence of malocclusion, which was reflected in the high normative need for orthodontic treatment. The opinion of patients regarding their expectations of orthodontic treatment should be valued. The students perceived a need for orthodontic treatment.

## RESUMO

**Objetivo:**

o objetivo do presente estudo foi avaliar a necessidade normativa de tratamento ortodôntico e a percepção dessa necessidade entre alunos de 11-14 anos de idade.

**Métodos:**

realizou-se um estudo transversal com uma amostra de 346 alunos selecionados aleatoriamente de escolas públicas e privadas. O Índice de Estética Dental (DAI) foi utilizado para avaliar a necessidade normativa de tratamento ortodôntico (NNTO). A percepção da necessidade de tratamento entre os estudantes foi avaliada por meio de um questionário previamente validado. Os dados foram analisados pelo teste qui-quadrado de Pearson (α = 5%).

**Resultados:**

a prevalência de má oclusão e NNTO foi de 65,6%. Da amostra, 73,7% relataram que precisavam de tratamento, 66,2% queriam tratamento ortodôntico e 62,7% estavam satisfeitos com sua estética dentária. Observou-se associação estatisticamente significativa entre o NNTO e a percepção da necessidade de tratamento entre os alunos (p < 0,001).

**Conclusão:**

o presente estudo demonstrou alta prevalência de má oclusão, o que se refletiu na alta necessidade normativa de tratamento ortodôntico. A opinião dos pacientes sobre suas expectativas de tratamento ortodôntico deve ser valorizada. Os alunos perceberam a necessidade de tratamento ortodôntico.

## INTRODUCTION

The need for orthodontic treatment can be determined by objective or normative evaluations. However, when based only on occlusal indices, the subjective perception of the patient has been ignored in the planning of treatment.^1^ Such indices are utilized to assess the severity of malocclusion and the normative need for orthodontic treatment (NNOT).^2^ ^-^ ^10^ The Dental Aesthetic Index (DAI) is a quantitative index which is recommended by the World Health Organization(WHO), and is considered the most frequently used tool in literature.^7^

The desire and perception of need of the population to use dental appliances should be considered.^11^ Aesthetic alterations to the face are easily perceived by individuals and can affect quality of life. Physical attractiveness is considered an important factor in personal relationships for young people.^12^ ^-^ ^13^ The perceived need for treatment by the patient or his/her family must be incorporated in clinical criteria.^1^ The aim of the present study was to evaluate the normative need for orthodontic treatment and the perception of this need by students aged 11-14 years.

## MATERIALS AND METHODS

This study was approved by the Ethics Committee on Human Research of the State University of Paraíba (CAAE 0100.0.133.000-11).

A cross-sectional study was conducted. The population studied consisted of students aged 11-14 years enrolled in public and private schools. The city of Campina Grande, Paraíba (PB), which has a Human Development Index of 0.721414, is divided into six health districts. The sample was collected based on health districts and clusters (schools), by stratification. Schools were randomly selected, with at least one public school and one private school chosen from each district. A random sample was selected in each school, proportional to the number of students per district.

The following were considered as eligibility criteria: (1) students aged 11-14 years enrolled in public and private schools in Campina Grande/PB; (2) absence of orthodontic treatment, before or at the time of the study; (3) authorization to participate in the study from parents/guardians; (4) cooperation of the student; (5) student had no neuropsychomotor deficiencies or deviations (mental, physical, sensory and behavioral order).

In the pilot study, 63 students were proportionally and randomly selected (public school, n = 23 and private school, n = 40). The prevalence of malocclusion was 63.5% and this value was used to calculate the final sample of the present study. The examinations were performed by a single examiner, who was an orthodontic specialist (Kappa = 0.816). Students who participated in the pilot study were not included in the main study. The reliability of the questionnaire was measured; for this, the test and re-test of the survey instrument was conducted after an interval of seven days. The agreement value was equal to 0.866.

The sample size was obtained by calculating the ratio estimation, considering a significance level of 95%, an error margin of 10% and a 63.5% prevalence of malocclusion (pilot study). A deff (design effect) of 1.5^15^ was used and 20% was added to the sample size to compensate for losses.^16^ The sample was calculated as 397 students, and there was a loss of 12.8%. The final sample included 346 students.

The DAI index was used to assess NNOT. This index includes the assessment of the following conditions: dentition (upper and lower tooth loss); space (crowding, spacing, diastema, anterior maxillary irregularity, anterior mandibular irregularity); occlusion (maxillary overjet, mandibular overjet, vertical anterior open bite and molar relationship).^7^ The intraoral physical exam was performed in natural light, with a flashlight used when needed as an auxiliary light. Periodontal probes (CPI type, Community Periodontal Index) were utilized to assess DAI index. Biosecurity standards were strictly followed.

A structured questionnaire, with epidemiological and socioeconomic data, was answered by students. Regarding the perception of the student in relation to the need for orthodontic treatment, three questions were asked: *“Do you think you need to use braces?”*, *“Do you want to use braces?”* and *“Are you satisfied with the aesthetics of your smile?”* ^16^

The collected data was recorded in the SPSS (Statistical Package for Social Sciences) for Windows software, version 20.0. Pearson’s chi-squared test was adopted for analysis. A significance level of 5% was adopted.

## RESULTS

The students were mostly female, 12 years old, whose parents had over 8 years of schooling, with a family income of up to two minimum salaries. The majority of students from public school were male, 13 years old, had parents with up to 8 years of schooling, and a family income of up to two minimum salaries. Most students of private school were 12 years old, female, with parents with more than 8 years of study, who also had a family income of two minimum salaries (Table 1). The prevalence of malocclusion was high (65.6%, n = 227). Most of the students had a need for elective treatment (Table 2). There was no statistical association between the need for orthodontic treatment and gender, age or type of school (Table 3). Most of the students believed they needed orthodontic treatment and desire to use braces and indicated that they were satisfied with their smiles (Table 4). Students of public school had a greater desire to use orthodontic braces than students of private schools (*p*< 0.001) (Table 5). An association was observed between the perceived need for orthodontic treatment by the students and the evaluation of a normative need for treatment. The students had the perception that they needed orthodontic treatment (*p*< 0.001) (Table 6).

**Table 1:** Characterization of the sample in terms of sociodemographic data.

Variables	Public		Private		TOTAL	
	n	%	n	%	n	%
Age (years)						
11	57	27.2	41	30.2	98	28.3
12	53	25.2	52	38.2	105	30.3
13	60	28.6	31	22.8	91	26.3
14	40	19	12	8.8	52	15.1
Gender						
Male	111	52.9	61	44.9	172	49.7
Female	99	47.1	75	55.1	174	50.3
Education of father**						
Illiterate	11	7.6	0	0	11	4.2
Up to 8 years	88	60.7	13	11.3	101	38.9
Over 8 years	46	31.7	102	88.7	148	56.9
Education of mother**						
Illiterate	8	4.9	0	0	8	2.7
Up to 8 years	89	53.9	12	9.8	101	35.1
Over 8 years	68	41.2	111	90.2	179	62.2
Household income**						
Up to 2 salaries *	171	92.5	67	56.8	238	78.5
Between 3 and 5 salaries *	11	5.9	32	27.1	43	14.2
More than 5 salaries *	3	1.6	19	16.1	22	7.3

* Minimum salary = U$ 311.00. ** the variable has missing data.

**Table 2:** Characterization of the sample regarding normative need for orthodontic treatment according to the DAI index.

Prevalence of malocclusion	DAI	Normative need for treatment	n	%
Yes	Defined malocclusion	Elective treatment	119	34,4
	Severe malocclusion	Highly desirable treatment	65	18,8
	Very severe malocclusion	Indispensable treatment	43	12,4
No	No abnormality	No or low need for treatment	119	34,4
TOTAL			346	100,0

**Table 3:** Association between normative treatment needs and demographic data in students aged 11-14 years.

Normative Need for Orthodontic Treatment (NNOT)									
Variables	No need		Elective		Desirable		Indispensable		
	n	%	n	%	n	%	n	%	X^2^ (p)^1^
Gender									
Male	59	17.1	61	17.6	36	10.4	16	4.6	3.64 (0.30)
Female	60	17.3	58	16.8	29	8.4	27	7.8	
School									
Public	71	20.5	73	21.1	40	11.6	26	7.5	0.09 (0.99)
Private	48	13.9	46	13.3	25	7.2	17	4.9	
Age									
11	24	6.9	37	10.7	22	6.4	15	4.3	11.93 (0.21)
12	36	10.4	41	11.9	14	4.0	14	4.0	
13	36	10.4	28	8.1	19	5.5	8	2.3	
14	23	6.7	13	3.8	10	2.9	6	1.7	

^1^ Pearson’s chi-squared test.

**Table 4:** Characterization of the sample regarding the perceived need for orthodontic treatment and dental aesthetics by students.

Variable	n	%
Do you think you need to use braces?		
Yes	255	73.7
No	89	25.7
No answer	2	0.6
Do you want to use braces?		
Yes	229	66.2
No	116	33.5
No answer	1	0.3
Are you satisfied with the aesthetics of your smile?		
Yes	217	62.7
No	127	36.7
No answer	2	0.6
TOTAL	346	100

**Table 5:** Association between the perception of need for orthodontic treatment and demographic variables.

Groups	Need**				χ²	Willingness to use**				χ²
	No		Yes		(p)^1^	No		Yes		(p)^1^
	n	%	n	%		n	%	n	%	
Gender										
Male	41	11.9	130	37.8	0.63 (0.42)	51	14.8	121	35.1	2.42 (0.11)
Female	48	14	125	36.3		65	18.8	108	31.3	
School										
Public	51	14.8	158	45.9	0.60 (0.43)	52	15.1	158*	45.7	18.88 (<0.001)
Private	38	11	97	28.3		64*	18.6	71	20.6	
Age (years)										
11	17	4.9	81	23.5	5.41 (0.14)	34	9.9	64	18.6	1.24 (0.74)
12	31	9	74	21.5		38	11	67	19.3	
13	25	7.3	65	18.9		30	8.7	61	17.7	
14	16	4.7	35	10.2		14	4.1	37	10.7	

* Statistically significant association (p < 0,05). ^1^ Pearson’s chi-squared test. **Variable has missing data.

**Table 6:** Association between the perception of need for orthodontic treatment and oral aesthetics among students and normative need for orthodontic treatment among students.

Normative need for orthodontic treatment									
Perception of student	No need		Elective		Desirable		Indispensable		χ²
	n	%	n	%	n	%	n	%	(p)^1^
Need for orthodontic treatment**									
No	40*	11.6	39*	11.3	8	2,3	2	0,6	23.44
Yes	79	23	78	22.7	57*	16.6	41*	11.9	(<0.001)
Desire to orthodontic treatment**									
No	45	13	39	11.3	20	5,8	12	3.5	1.82
Yes	74	21.5	79	22.9	45	13	31	9	(0.61)
Perception of aesthetics of smile**									
Unsatisfactory	36	10.5	44	12.8	30	8,7	17	4.9	4.93
Satisfactory	82	23.8	75	21.8	34	9,9	26	7.6	(0.17)

*Statistically significant association (p < 0,05). ^1^ Pearson’s chi-squared test. **The variable has lost data.

## DISCUSSION

The adoption of the DAI index for conducting studies like this is based on the fact that it is recommended by the WHO and is classically used in epidemiological surveys to assess the presence of malocclusion and the normative need for orthodontic treatment.^8^ ^,^ ^13^ ^,^ ^17^ ^-^ ^19^ The prevalence of malocclusion and the normative need for treatment was high in this study. The need for treatment was highly desirable or essential among almost half the students. This data is consistent with other studies in literature.^6^ ^,^ ^19^ ^,^ ^20^ There was no association between the normative need for orthodontic treatment and the age and gender of the students, findings that were in agreement with the results of Marques et al^16^. The high prevalence of a need for orthodontic treatment among low-income populations can be explained by difficulties in access to health services.^21^

Decisions regarding orthodontic treatment should include the opinions of adolescents and their relatives.^1^ The perception of adolescents regarding their need for treatment is a success factor for such treatment.^1^ ^,^ ^12^ In the present study a high percentage of students reported needing orthodontic treatment. Cases of malocclusion can affect aesthetics and lead to the search for orthodontic treatment. Such need can also be influenced by the desire to appear attractive due to the search for greater self-esteem.^4^ ^,^ ^20^ The present study observed that most students were satisfied with their smiles, data which disagreed with the study of Winnier et al^19^.

Public school students exhibited a greater desire to use orthodontic braces than private school students. This situation can be explained by the fact that students of public schools do not have access to this type of treatment, which is considered very expensive. This data is consistent with literature.^1^ Peres et al^18^ reported an association between the perception of dental aesthetics and gender. Girls seem to value beauty and attractiveness more than boys. This association was not observed by Badran^22^ or in the present study.

An association was observed between the perceived need for orthodontic treatment by the student and the evaluation of a normative need for treatment. These findings were reported by other studies.^16^ ^,^ ^22^ For Claudino and Traebert,^23^ there was a strong correlation between the presence of malocclusions and the perception of dental aesthetics requiring orthodontic treatment observed by these individuals.

Nowadays, teenagers have developed the awareness and perception to identify their oral problems. However, this factor has not been included in the definition of the normative clinical criteria for malocclusion. Patients can have a more positive outlook on malocclusions, and often the opinions of professionals overestimate the need for treatment.^24^ There was a correlation in this study between the normative need for treatment and the perception of this need by students. However, the association between the normative need for treatment and the perception of the aesthetics of the smile was not statistically significant. These results differed from data found by Winnier et al^19^ who identified a strong statistical association between these variables.

## CONCLUSIONS

The present study demonstrated a high prevalence of malocclusion, which reflects a high normative need for orthodontic treatment. There is a clear need to value the opinions of patients regarding their expectations of orthodontic treatment.
